# Leptin Concentrations Determine the Association between High-Sensitivity C-Reactive Protein Levels and Body Mass Index in Prepubertal Children

**DOI:** 10.3390/nu15102388

**Published:** 2023-05-19

**Authors:** Claudia Vales-Villamarín, Olaya de Dios, Iris Pérez-Nadador, Teresa Gavela-Pérez, Leandro Soriano-Guillén, Carmen Garcés

**Affiliations:** 1 Lipid Research Laboratory, IIS-Fundación Jimenez Diaz, UAM, 28040 Madrid, Spain; claudia.vales@quironsalud.es (C.V.-V.); olayadedios@gmail.com (O.d.D.); iris.perezn@quironsalud.es (I.P.-N.); 2 Department of Pediatrics, IIS-Fundación Jimenez Diaz, UAM, 28040 Madrid, Spain; tgavela@fjd.es (T.G.-P.); lsoriano@fjd.es (L.S.-G.)

**Keywords:** children, hs-CRP, body mass index, leptin

## Abstract

Obesity is associated with the presence of low-grade inflammation even during childhood. The dysregulation in the secretion of adipokines, such as leptin, which occurs in obesity states, could be associated with an increase in inflammatory factors already at an early age. In this cross-sectional study, we aimed to investigate the role of leptin levels in the association between body mass index (BMI) and high-sensitivity C-reactive protein (hs-CRP) in healthy schoolchildren. Leptin and hs-CRP levels were analyzed in two pediatric cohorts comprising 684 prepubertal children and 763 adolescents. hs-CRP concentrations correlated significantly with BMI and leptin levels in prepubertal males and females as well as in adolescents. However, after adjusting for leptin concentration, no significant correlation was observed between hs-CRP and BMI in prepubertal children, while the correlations remained significant in adolescents. The same differences were observed when analyzed BMI according to hs-CRP tertile after adjusting for leptin; mean BMI was not significantly different between hs-CRP tertile in prepubertal children but was significantly different in adolescents. In conclusion, the fact that leptin concentrations determine the association of BMI with hs-CRP levels in prepubertal children, but not in adolescents, suggests a role for leptin in low-grade inflammation at early ages, while other factors seem to contribute to hs-CRP levels later in life.

## 1. Introduction

Obesity was related to the presence of a systemic low-grade inflammatory state, even during childhood [1,2]. C-reactive protein (CRP) is an acute-phase inflammatory reactant, and high-sensitivity C-reactive protein (hs-CRP) concentration is currently used as an indicator of systemic inflammation and cardiovascular risk [3]. Obesity was related to increased concentrations of CRP, not only in adults [4,5] but also in children [5,6]. Findings from the National Health and Nutrition Examination Survey [7] evidenced that body mass index (BMI) was the best predictor of CRP concentrations in young children and adolescents [7].

The dysregulation of adipocytokine secretion that occurs in obesity states was consistently associated with an increased secretion of a number of inflammatory factors, as these adipocytokines that are secreted from adipose tissue are involved in inducing the production of CRP [8]. Adipose tissue is related with the release of the pro-inflammatory cytokine Interleukin-6 (IL-6) which triggers the synthesis by the liver of CRP [9]. Both IL-6 and CRP levels were associated with the obesity characterized by an increase infat mass in normal weight participants [10]. IL-6 was related to pathologies such as atherosclerosis, showing an important role in the vascular system [11].

Among the adipokines secreted by adipose tissue, leptin was consistently associated with the development of obesity-related complications [12]. It was suggested that leptin resistance may be a link between inflammation and metabolism in obesity-related disorders [13]. Leptin levels were positively associated with CRP concentrations in adults [14,15], although the association seems to depend on sex [14]. Studies in children are scarce [16,17], but both leptin and CRP levels were shown to be associated with metabolic syndrome in children [18]. We previously described a significant positive association between hs-CRP levels and leptin concentrations in two cohorts of Spanish children [19]. More recently, our group reported that leptin concentrations influence the association of obesity with parameters related to insulin resistance, such as non-esterified NEFA levels [20].

Using a cross-sectional epidemiologic design, the aim of the present study was to analyze the potential influence of leptin levels in the association between body mass index and hs-CRP concentrations in two cohorts of children, prepubertal children (age 6 to 8 years) and adolescents between the ages of 12 and 17 years.

## 2. Materials and Methods

### 2.1. Participants

We analyzed two population-based samples comprising 684 prepubertal children (6-to-8-year-olds) and 763 adolescents with ages between 12 and 17 years. This was a sub-study of the Four Provinces Study, a cross-sectional study designed to analyze cardiovascular risk factors in children. A more detailed information on the study design is available in previous publications [19]. The selection of children was carried out by random sampling, by clusters of schools, stratified by sex and type of school (public or private ownership). Sampling was carried out in two stages. In the first, the schools were selected from the lists available in the territorial delegations of the Ministries of Education. In the second, the classrooms and students were selected.

The study protocol was approved by the Clinical Research Ethics Committee of the Instituto de Investigación Sanitaria Fundación Jiménez Díaz (IIS-FJD). This investigation was carried out in accordance with the principles of the Declaration of Helsinki and subsequent reviews, as well as the prevailing Spanish legislation on clinical research in human participants [19].

Children who, according to parents’ reports, suffered from metabolic, endocrine, hepatic, or renal disorders were excluded, due to possible alterations in the values of the variables of interest [19]. Special attention was paid to exclude children suffering from an acute infection from our study.

### 2.2. Collection of Information and Study Variables

Oral presentations of the study were carried out at each center selected in the sample and a letter was sent to the parents of the children invited to participate in the study with information on the objectives and procedures of the same. Then, all parents were required to give written consent for their children to be included.

In each center, the information was collected by a field team made up of a doctor, a nurse and a group of people trained in completing the food consumption frequency questionnaire, obtaining information from the mothers of the children or the people in charge of preparing the menu.

#### 2.2.1. Anthropometric Data

Measurements (weight and height) were taken with children barefoot and wearing light clothing as reported [19]. Height was measured to the millimeter using a portable stadiometer, and weight was recorded to the nearest 0.1 kg using a standard electronic digital scale. Body mass index (BMI) (weight in kilograms divided by height in meters squared, kg/m^2^) was calculated from these parameters [19].

#### 2.2.2. Biochemical Data

Venous blood samples were obtained early in the morning, after a 12 h fasting period, by venipuncture into Vacutainer tubes containing EDTA-Na2 as anticoagulant. Then, samples were placed on ice bath, and centrifuged at low speed. The plasma was separated and frozen for future biochemical determinations.

Plasma leptin levels were determined by ELISA using a commercially available kit (Leptin EIA-2395, DRG Instruments GmbH, Marburg, Germany) [19]. C-reactive protein levels were measured using an hs-CRP ELISA kit (CRP High Sensitivity SK00080-02, Aviscera Bioscience, Inc., Santa Clara, CA, USA) [19]. hs-CRP levels were measured with a detection limit of 0.15 mg/L. Children with hs-CRP levels of 10 mg/L or above were excluded to avoid the influence of acute infection.

#### 2.2.3. Nutritional Information

Diet information was available in the cohort of pre-pubertal children of our study. As reported previously [21], food and nutritional information was obtained through a food consumption frequency questionnaire, initially developed in adults, and adapted to the school population for this study by modifying the list of foods and the portions consumed. Based on the Spanish food composition tables, a food consumption frequency conversion program was designed that provides a base with the annual frequency of food consumed and the daily frequency of consumption of the nutrients. Thus, nutrients and total caloric intake were estimated [21].

### 2.3. Statistical Analysis

Statistical analyses were performed using the SPSS software package, version 26.0 (IBM, New York, NY, USA) and the GraphPad Prism statistical software (San Diego, CA, USA, Version 8). The Kolmogorov–Smirnov test was used to determine whether the variables under study were normally distributed. Spearman correlation and partial correlation analyses were performed to evaluate the association between the variables under study in both cohorts of children. Analysis of variance (ANOVA) and the Games–Howell post hoc test were used to compare body mass index and leptin levels by hs-CRP tertile. Univariate analyses of variance were performed to analyze the associations between body mass index and hs-CRP by age group and sex after adjusting for leptin levels.

## 3. Results

Our population comprised 327 6-to-8-year-old males and 357 females of the same age as well as 358 males and 405 females within a range of the ages between 12 and 17 years. The average age of the participants in our study was 7.2 ± 0.6 years in the prepubertal group and 14.4 ± 0.8 years in the group of adolescents. No significant differences between sexes were observed.

The values of hs-CRP were below or equal to the detection limit in 33% of 6–8-year-old children and in 26% of 12–16-year-old children [19].

A significant positive correlation was found between body mass index and leptin levels (*p* < 0.001), as well as between body mass index and hs-CRP concentrations in both males (*p* < 0.01) and females (*p* < 0.001) of the younger cohort. However, the correlation between body mass index and hs-CRP was not significant in these children after adjusting for leptin levels (Table 1). In 12-to-17-year-old boys and girls, we also observed significant correlations (*p* < 0.001) for body mass index with leptin and hs-CRP concentrations. In this group, the correlations between body mass index and hs-CRP remained significant after adjusting for leptin concentration (Table 1).

**Figure 1:**
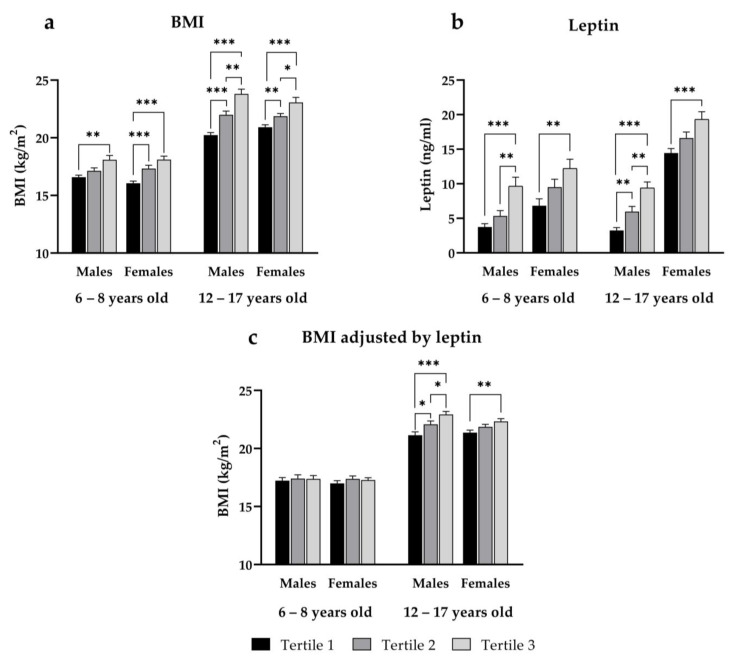
BMI (**a**), leptin levels (**b**) and BMI adjusted for leptin levels (**c**) according to hs-CRP concentration tertile in males and females of both age groups. Data shown as mean and standard error. *p*-value: * *p* < 0.05; ** *p* < 0.01; *** *p* < 0.001.

To further confirm our findings, and due to the skewed distribution of hs-CRP data, mean body mass index values and leptin levels were compared among hs-CRP tertiles depending on age and sex (Figure 1a,b, respectively). The levels of hs-CRP in each tertile group were as follows: in boys with ages between 6 and 8 years, tertile 1 (hs-CRP ≤ 0.15 mg/L), tertile 2 (hs-CRP between 0.16 and 0.58 mg/L), tertile 3 (hs-CRP ≥ 0.60 mg/L); in girls with ages between 6 and 8 years, tertile 1 (hs-CRP ≤ 0.15 mg/L), tertile 2 (hs-CRP between 0.16 and 0.59 mg/L), tertile 3 (hs-CRP ≥ 0.60 mg/L); in boys with ages between 12 and 17 years, tertile 1 (hs-CRP ≤ 0.16 mg/L), tertile 2 (hs-CRP between 0.17 and 0.55 mg/L), tertile 3 (hs-CRP ≥ 0.56mg/L) and in girls with ages between 12 and 17 years, tertile 1 (hs-CRP ≤ 0.16 mg/L), tertile 2 (hs-CRP between 0.17 and 0.55 mg/L), tertile 3 (hs-CRP ≥ 0.56 mg/L). We observed a gradual increase in body mass index across hs-CRP concentration category in 6-to-8-year-old and 12-to-17-year-old boys and girls (Figure 1a), with significant (*p* < 0.001) differences between tertiles at both ages. Leptin levels also varied significantly across hs-CRP categories in both prepubertal boys (*p* < 0.001) and girls (*p* < 0.01) and in 12-to-17-year-old boys (*p* < 0.001) and girls (*p* < 0.01), showing a significant increase across hs-CRP tertiles (Figure 1b).

On univariate ANOVA, after adjusting for leptin, mean body mass index remained significantly different across hs-CRP tertiles in 12-to-17-year-old boys (*p* < 0.001) and girls (*p* < 0.01) (Figure 1c) but showed no significant differences between hs-CRP tertiles in either boys or girls between 6 and 8 years of age (Figure 1c).

We previously described an inverse association of fruit and vegetable intakes with hs-CRP concentrations in the prepubertal females; thus, we decided to analyze the potential influence of these intakes on the association between body mass index and hs-CRP concentrations in the cohort of 6-to-8-year-old children of our study. No significant results were found.

## 4. Discussion

Previous research on cardiovascular risk factors in Spain using the same cohort of prepubertal children described here found that leptin concentration influenced the association between obesity and features of insulin resistance [20]. In the present study, we aimed to analyze the potential influence of leptin on the association between body mass index and CRP, by means of analyzing high sensitivity CRP (hs-CRP), a useful marker of low-grade inflammation. Two cohorts of children were analyzed: prepubertal (age 6 to 8 years) and adolescent participants (12 to 17 years). In the older children, the association between body mass index and hs-CRP remained significant for both sexes after adjusting for leptin concentrations; however, the correlation disappeared when adjusting for leptin levels in 6-to-8-year-old children.

Leptin seems to regulate proinflammatory immune responses and was related to CRP secretion [22]. CRP is synthesized in the liver when stimulated by interleukin-6 (IL-6), a proinflammatory cytokine released from adipose tissue [9]. Although the liver is the primary site of CRP synthesis [22], it was suggested that adipose tissue may be an additional source of CRP [23,24,25].

Leptin levels were associated with IL-6 in a study investigating the relationship of these adipocytokines with overweight and obesity in participants at different ages [26]. Interestingly, however, the correlation between leptin and IL-6 plasma levels was observed only in young individuals, while in adults, the waist-to-height ratio was the main predictor of IL-6 levels. The authors suggested that leptin may determine IL-6, which stimulates CRP, mainly in juveniles [26].

Along with these previous reports, our findings reporting that the correlation between body mass index and hs-CRP disappeared when we adjusted for leptin levels in prepubertal children, while the association remained significant after the adjustment in adolescents, may indicate an important role of leptin in the earlier phases of obesity-related inflammation. The role of leptin in the pathogenesis of inflammatory responses was established [27,28], and when interpreted in light of the report by Stelzer I et al. [26], our findings highlight the contribution of leptin in the induction of low-grade inflammation in young children.

The seemingly unessential role of leptin in older children suggests the existence of other mediators of hs-CRP levels. CRP was found to be increased in both liver and adipose tissue in obese patients [24]. It was assumed that obesity-related inflammation is mainly associated with the secretion of adipocyte-specific IL-6-type cytokines, which stimulate hepatic production of CRP [29,30]. However, macrophage infiltration in adipose tissue is also related to the inflammatory process [31]. A different body composition (amount and distribution of body fat) was observed in children depending on age. Fat quantity and distribution substantially influence CRP [32]. Furthermore, it was shown that the number of adipose tissue macrophages correlates positively with age [33]. Thus, we could hypothesize that a slight increase in the presence of macrophages with age could explain our findings to an extent. Additionally, the fact that IL-6 is involved in the remodeling of the vascular system, which is likely increased in children, should be taken under consideration.

A relationship between diet and hs-CRP concentrations was previously reported by our group in adults [34] as well as in the prepubertal cohort of children used in the current study [21]. In the study in children, we described an association of lower hs-CRP levels with higher vegetable and fruit intakes [21]. In the study in adults, we described the relationship hs-CRP levels with adherence to a Mediterranean diet [34]. However, no influence of nutritional intake was observed on the association between body mass index and hs-CRP levels in the prepubertal children of our study.

Unfortunately, data on IL-6 concentrations were lacking from our study, as we were unable to measure this parameter. As a result of this primary limitation to our study, further research taking into consideration IL-6 levels is needed to clarify these findings. An additional important limitation is the lack of information on fat mass, which would have enabled us to compare this aspect between the two pediatric cohorts.

Further research is required to clarify the role of leptin in inflammation.

## 5. Conclusions

The association of body mass index with hs-CRP concentrations in prepubertal children seems to be mediated by leptin levels, while the increased hs-CRP concentrations associated with higher body mass index in adolescents appears to remain independent of plasma leptin concentrations and may be related to other factors.

## Figures and Tables

**Table 1:** Correlation analysis between body mass index and hs-CRP and leptin, and between body mass index and hs-CRP adjusted for leptin in 6-to-8-year-old and 12-to-17-year-old boys and girls.

	Ages 6 to 8 Years			
	BMI Non—adjusted		BMI Adjusted for leptin	
	Males	Females	Males	Females
hs-CRP	0.183 **	0.280 ***	0.151	−0.055
Leptin	0.632 ***	0.663 ***	-	-
	**Ages 12 to 17 Years**			
	BMI Non—adjusted		BMI Adjusted for leptin	
	Males	Females	Males	Females
hs-CRP	0.367 ***	0.215 ***	0.130 *	0.119 *
Leptin	0.561 ***	0.643 ***	-	-

* *p* < 0.05; ** *p* < 0.01; *** *p* < 0.001.

## Data Availability

Data are contained within the article.
